# Greater than the sum of its parts? Modelling population contact and interaction of cultural repertoires

**DOI:** 10.1098/rsif.2017.0171

**Published:** 2017-05-03

**Authors:** Nicole Creanza, Oren Kolodny, Marcus W. Feldman

**Affiliations:** 1 Department of Biological Sciences, Vanderbilt University, Nashville, TN 37235-1634, USA; 2 Department of Biology, Stanford University, Stanford, CA 94305, USA

**Keywords:** cultural accumulation, migration, population structure, connectivity, archaeology

## Abstract

Evidence for interactions between populations plays a prominent role in the reconstruction of historical and prehistoric human dynamics; these interactions are usually interpreted to reflect cultural practices or demographic processes. The sharp increase in long-distance transportation of lithic material between the Middle and Upper Palaeolithic, for example, is seen as a manifestation of the cultural revolution that defined the transition between these epochs. Here, we propose that population interaction is not only a reflection of cultural change but also a potential driver of it. We explore the possible effects of inter-population migration on cultural evolution when migrating individuals possess core technological knowledge from their original population. Using a computational framework of cultural evolution that incorporates realistic aspects of human innovation processes, we show that migration can lead to a range of outcomes, including punctuated but transient increases in cultural complexity, an increase of cultural complexity to an elevated steady state and the emergence of a positive feedback loop that drives ongoing acceleration in cultural accumulation. Our findings suggest that population contact may have played a crucial role in the evolution of hominin cultures and propose explanations for observations of Palaeolithic cultural change whose interpretations have been hotly debated.

## 1. Introduction

Long-distance hominid mobility, which probably correlates with inter-population connectivity, can be inferred from various aspects of the archaeological record; for example, transportation of material and artefacts over distances greater than 100 km occurred sporadically in the Middle Palaeolithic and regularly in the Upper Palaeolithic [1]. This feature of the Upper Palaeolithic revolution is usually attributed to demographic processes, changes in subsistence strategies or other cultural shifts [1,2]. We suggest that inter-population connectivity may be more than a reflection of cultural advancement: it may have been critical in *driving* such change. In this study, we explore the cultural dynamics that may result from population contact.

Connectivity within and between populations has been proposed, in theoretical and anthropological studies, to dramatically influence cultural evolution [3–9]. An experimental human-interaction study showed that groups produce more complex artefacts than individuals acting alone [10], and several anthropological studies and evolutionary models suggest a relationship between group size and technological complexity (e.g. [11–13]). In the workplace, innovations appear more often when members of different groups interact [14]. Further, experimental groups that independently accumulated traits and then combined their knowledge made successful innovative combinations not observed in fully connected groups [15]. Similarly, a recent model simulated contact between populations with a continuum of mobility strategies, from remaining near a home base to constantly moving, with no home base [16]; the results suggested that intermediate strategies, which might ensure both regular contact with new populations and enough contact time to accurately transmit information, maximized cultural transmission across population boundaries. From their archaeological analyses, Stiner & Kuhn [17] suggested that the connectedness of the Upper Palaeolithic could have stabilized technological volatility, decreasing risk and increasing demographic robustness. Along the same lines, Hovers & Belfer-Cohen [18] proposed that population interconnectedness prevents local loss of culture, and that the Middle Palaeolithic record reflects a pattern of cultural extinction and re-invention, stemming from instability of transmission networks. These empirical and theoretical studies suggest that modelling the effect of inter-population interactions on overall cultural complexity may be useful in interpreting the archaeological record of hominid culture.

One of the apparent features of the time trajectory of culture is that it includes periods of relative stasis that are separated by bursts of cultural accumulation; these increases can differ in both time scale and magnitude [19–22]. Previous explanations for punctuations in the archaeological record have invoked a cultural reaction to such factors as genetic/cognitive change in hominids or environmental change that alters the population's cultural steady state [21,23–27]. In a previous study, we proposed that independent innovation processes can explain cultural bursts: if a cultural advance facilitates associated innovations and novel trait combinations, then a purely cultural mechanism can trigger a cascade of related innovations and punctuated cultural bursts [28].

An alternative driver of punctuation could be sudden changes in the parameters of cultural evolution, such as those brought about by modification of the *biological carrying capacity*, the number of individuals that the available resources can support [29]. Thus, a cultural trait, for example a tool or practice related to agriculture [30,31], could increase food availability and the biological carrying capacity. The resulting population growth might correspond to an increased cultural repertoire, as predicted by experiments, some cross-cultural analyses and cultural–evolutionary models [11–13,32–40]. Notably, these carrying-capacity-altering cultural shifts can lead to much greater cultural accumulation [29] than those induced by a cascade of related innovations in the model of [28].

Most models of cultural evolution consider the spread of existing traits, but only a handful explicitly model the innovation processes that underlie the origin of new traits [28,41,42]. Some models have addressed the effect of population structure; for example, migration among subpopulations may affect the population's cultural diversity [37,40,43] and the accumulation of errors [43]. Further, migration among subpopulations could affect the cultural repertoire, both because cultural loss is less likely with access to more cultural models [12] and because rare innovations are more likely to spread throughout the population [44]. In [44], migration and population size had a greater effect on pre-equilibrium dynamics than on the cultural equilibrium of a population, but this analysis examined the skill level of a finite cultural repertoire as opposed to additions to a potentially limitless cultural repertoire.

To study the effects of inter-population contact on cultural dynamics, we develop a theoretical framework that considers jointly cultural contact, innovation and modifiers of biological carrying capacity. Here, populations innovate and accumulate cultural traits independently, and individuals migrate between populations bringing the core technologies invented in their original population, which facilitates cultural change. In addition, technologies can be combined to form new tools, and the many novel combinations that become possible following a migration event may potentially trigger a burst of innovations. As in real-life human cultures, the potential number of cultural traits in our model is theoretically unlimited.

By considering the effects of both population size and population structure on cultural accumulation, our model addresses human experimental and archaeological data. In particular, our model suggests that large-scale punctuation in the archaeological record can result from an increase in inter-population connectivity. We explore the possibility that cultural contact is a primary driver of rapid cultural change and characterize patterns that would emerge under different migration regimes.

## 2. The model

We extend the model of Kolodny *et al.* [28] to include multiple populations whose cultures independently innovate and evolve. We simulate the effects of migration and cultural interactions between these populations. In the model, we assume that each individual has some probability of innovating and of migrating, so the overall rates of innovation and migration in the population are proportional to population size. Similarly, we assume that cultural traits are more susceptible to loss when fewer people know them, so the overall rate of cultural loss is inversely related to population size. Finally, we assume that certain rare innovations, such as those that increase the food supply, can increase carrying capacity and thus affect population size. Although one cannot generalize to all human populations from a single model, a body of empirical and theoretical literature supports these assumptions (e.g. [11,13,30,34–38,45]).

In the model, three interacting processes contribute to human tool innovation, as in [28]. The first process produces ground-breaking large-scale innovations, or *lucky leaps*, which occur with probability *P*_lucky_ per individual per time step. Each lucky leap innovation facilitates two other tool innovation processes. First, a number of tools are made useful by each lucky leap; these are termed *toolkit innovations*. There are *L* toolkit innovations associated with each lucky leap, where *L* is sampled from a uniform distribution *U*(1,11).

Lucky leap innovations can also combine with other lucky leap innovations to produce *innovative combinations*, which are useful to the population with probability *P*_combUseful_. With only lucky leaps allowed to combine, this relatively conservative combination scheme represents the notion that ground-breaking ideas are often widely applicable to other existing technologies. For simplicity, we assume that all potentially useful combinations and toolkit tools are innovated immediately upon the lucky leap's invention, which is equivalent to the assumption that an individual tests more than one combination per time step.

Several models represent cultural traits as skills and track variation in individual skill levels [12,13,35,44], and others track the presence of traits in individual cultural repertoires [34,37,40,46]; in these studies, transmission between individuals is explicitly modelled, and cultural complexity increases with population size. Here, we build upon these findings to simplify the transmission process: we track the population-level presence of traits, as in [36,38], and we assign a probability that a trait will arise and spread in the population rather than focusing on individual-level transmission processes.

Finally, at each time step, tools may be stochastically lost due to drift. Because the rate of cultural loss is likely to decrease as the population grows [13], we scale the loss parameter, *P*_SpontLoss_, by the population size: *P*_SpontLoss_*/N*. This loss probability encapsulates numerous possible loss processes, including failed transmission between individuals and fluctuating trait frequencies that may decrease to extinction by chance. In reality, a certain trait's probability of loss depends on many factors, including its ease of transmission, effect on biological fitness and usefulness in the current environment [28]; for simplicity, we use the same probability of loss for all traits. (A similar approximation is found in [36] and the electronic supplementary material of [38], where agent-based transmission of knowledge is taken into account.) This rate of stochastic trait loss is a useful first approximation that captures how loss might scale with population size and how even important traits can be lost [13].

When a lucky leap tool is lost, the toolkit and combination tools associated with it are also lost. Toolkit innovations and combination tools, however, can be individually lost without affecting other tools. These tools may also be re-invented in later time steps, if the lucky leap innovations with which they were associated remain in the population. This occurs with toolkit and combination tools, respectively, with probabilities *P*_Toolkit_ and *P*_Combine_ per individual per time step.

In these agent-based stochastic simulations, each process occurs with a given probability per time step; thus, each run of the stochastic simulation is unique. In the electronic supplementary material, we also give equations for the *expected* effect of each process under simplifying assumptions.

The framework outlined above is sufficient to produce punctuated bursts of innovations after periods of stasis, as a lucky leap innovation can facilitate the relatively rapid addition of combinations and toolkit innovations [28]. When tools can be lost as well as added, the mean number of each type of tool (*n*_lucky_, *n*_toolkit_, *n*_comb_) approaches a steady state (derived in the electronic supplementary material):2.1
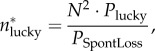
2.2
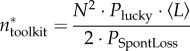
2.3
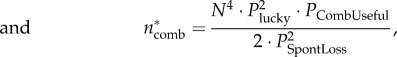
where *N* is the population size and 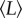 is the mean number of toolkit innovations associated with each lucky leap tool.

**Figure 1.**
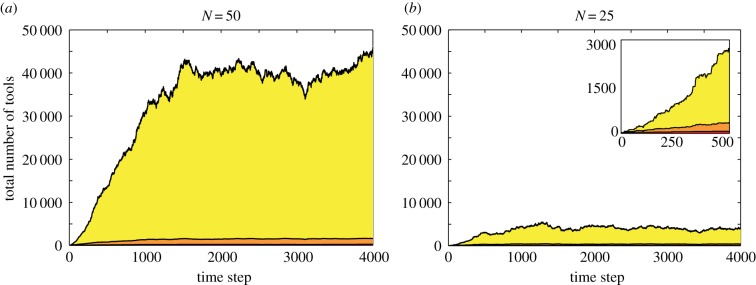
The tool repertoire size of a population of size 2*N* (*a*) is much larger than the sum of two populations of size *N* (*b*). In this example, a population has approximately 40 000 tools, whereas the same population divided into two disconnected subpopulations has approximately 8000 tools at steady state. In (*b*), one population's cultural trajectory is shown. In both panels, red indicates lucky leaps (visible at the bottom of the inset of (*b*)), orange indicates toolkit innovations and yellow indicates combination tools. Other parameters in (*a*) populations *=* 1, *N* = 50, *P*_lucky_ = 0.08, *P*_combUseful_ = 1, *P*_SpontLoss_ = 0.08, *P*_migrate_ = 0; (*b*) populations *=* 2, *N* = 25, *P*_lucky_ = 0.08, *P*_combUseful_ = 1, *P*_SpontLoss_ = 0.08, *P*_migrate_ = 0.

**Figure 2.**
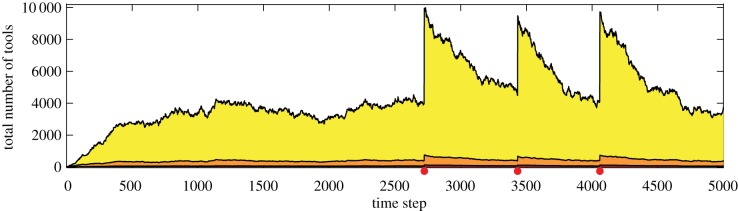
The effect of rare migration on cultural repertoire size. For *t* = 0 to *t* = 2000, there is no migration (*P*_migrate_ = 0); after *t* = 2000, migration is possible but very rare (*P*_migrate_ = 0.000025). One population's cultural trajectory is shown. Migration events (red dots on the *x*-axis) represent the arrival of a new individual to the population. Following the initial burst of culture driven by the combinations between the existing tools and those introduced by migration, there is a gradual decay back to the steady state. Other parameters: populations *=* 2, *N* = 25, *P*_lucky_ = 0.08, *P*_combUseful_ = 1, *P*_SpontLoss_ = 0.08.

**Figure 3.**
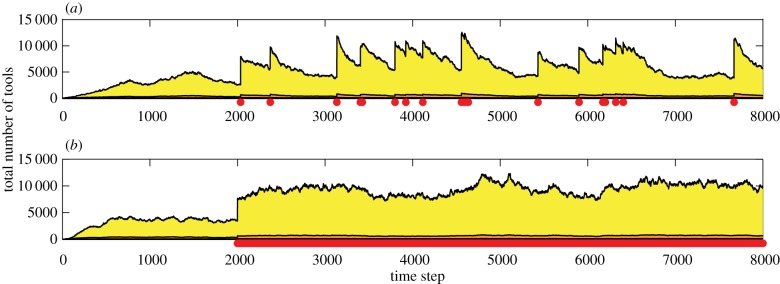
The effect of frequent migration on cultural repertoire size. As in figure 2, *P*_migrate_ = 0 for *t* = 0 to *t* = 2000; after *t* = 2000, *P*_migrate_ = 0.0001 in (*a*) and *P*_migrate_ = 0.4 in (*b*). Each panel illustrates one population's cultural trajectory. In (*a*), migration events are indicated by red dots on the *x*-axis; in (*b*), these events occur so frequently (more than once per time step) that the dots are individually indistinguishable. As the overall migration rate increases, the cultural repertoire does not return to the original steady state between migration events; thus, migration effectively elevates the cultural steady state of the population. Other parameters (*a,b*): populations *=* 2, *N* = 25, *P*_lucky_ = 0.08, *P*_combUseful_ = 1, *P*_SpontLoss_ = 0.08.

**Figure 4.**
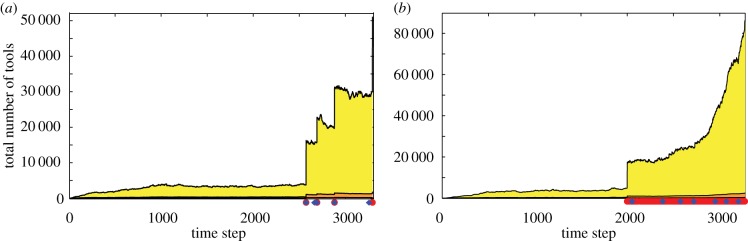
The effect of frequent migration and changes in carrying capacity on cultural repertoire size. *P*_migrate_ = 0 for *t* = 0 to *t* = 2000; after *t* = 2000, *P*_migrate_ = 0.0001 and *P*_IncreaseCarryingCapacity_ = 0.0001 in (*a*), and *P*_migrate_ = 0.005 and *P*_IncreaseCarryingCapacity_ = 0.0005 in (*b*). Each panel illustrates one population's cultural trajectory. Red dots indicate migration events, and blue diamonds indicate the origin of innovations that trigger growth of carrying capacity. When migration is rare and innovations alter the carrying capacity relatively rarely, the cultural trajectory appears punctuated (*a*); changes to carrying capacity frequently occur following migration events due to the burst of new combinations that they induce. When migration is more frequent, innovations alter the capacity more often and the cultural repertoire increases rapidly without approaching a steady state (*b*). Other parameters (*a,b*): populations *=* 2, *N* = 25, *P*_lucky_ = 0.08, *P*_combUseful_ = 1, *P*_SpontLoss_ = 0.08. Each increase in carrying capacity (blue diamonds) is by a factor of between 1.1 and 1.2; by the end of the simulation shown, the population in panel (*a*) had reached *N* = 54, and the population in panel (*b*) reached *N* = 65.

**Figure 5.**
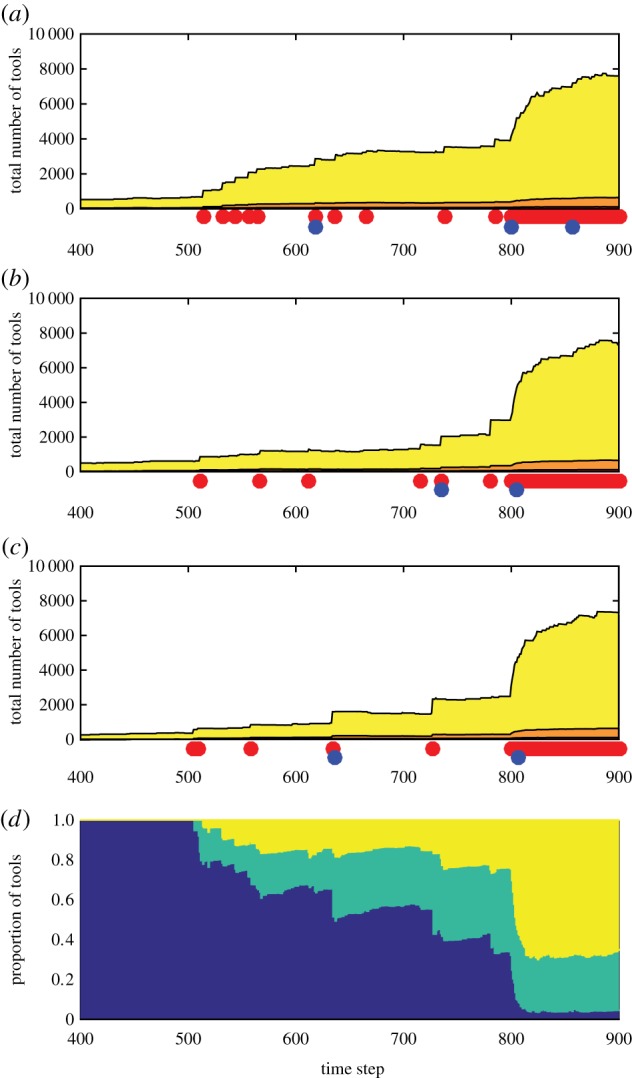
Co-development of partially connected populations. Panels (*a–c*) show cultural dynamics in three contemporaneous populations (*N*_1_ *=* *N*_2_ *=* *N*_3_ *=* 25; colour scheme as in previous figures). Panel (*d*) shows the fraction of cultural overlap of combination tools among populations: the mean fraction of tools in each population that are unique to that population (blue), the mean fraction of combination tools that are shared with one other population (cyan), and the mean fraction of combination tools that are common to all three populations (yellow). Each population's culture is unique (*P*_migrate_ = 0) until *t* = 500, and cultural complexity is near steady state for long periods of time. From *t* = 500 to *t* = 800, *P*_migrate_ = 0.0004. During this phase, partially coordinated cultural change occurs, while each population remains culturally distinct: migration events (red dots) drive punctuated increases in cultural complexity (each migrant introduces into the new population each of the core technologies from its original population with probability *f*_migrant_ = 0.3; the new combination tools that become possible drive the increase in cultural complexity), and inventions that increase biological carrying capacity spread quickly (blue dots, (*a–c*)). Overall repertoire sizes increase in all populations by similar orders of magnitude, while cultural overlap of combination tools increases gradually, but with a significant fraction of each population's repertoire remaining unique (*d*). At *t* = 800, *P*_migrate_ is increased to 0.04. This more frequent migration leads to a state reminiscent of a single large population, driving overall cultural repertoire sizes upwards sharply (*a–c*) and effectively near-homogenizing the populations’ cultures (*d*). Other parameters (*a–d*): *P*_IncreaseCarryingCapacity_ = 0.01, *P*_lucky_ = 0.02, *P*_SpontLoss_ = 0.02. Every increase in biological carrying capacity (blue dots) is by a factor of between 1.1 and 1.2, leading the populations to increase by the end of the simulation from *N*_1_ *=* *N*_2_ *=* *N*_3_ *=* 25 to *N*_1_ *=* 36*, N*_2_ *=* 39 and *N*_3_ *=* 32*.*

Here, we extend this framework [28] to include the effects of population size and cultural contact. First, we implement multiple simulations of the model simultaneously, generating independent populations that undergo innovation and cultural evolution. (The present framework can simulate many interacting populations with qualitatively similar results; figures 1–5 display two or three populations for ease of visualization.) Then, individuals migrate between populations with probability *P*_migrate_ per time step. An individual enters a new population carrying with it some fraction, *f*_migrant_, of the full repertoire of core technologies in its original population, i.e. its repertoire of lucky leap innovations and their associated toolkits. We explored the dynamics of population-level subdivision of knowledge in [28]; for simplicity in this study, in figures 1–4 we set *f*_migrant_ = 1, i.e. each migrant carries its originating population's full cultural repertoire; this does not influence the results qualitatively. Following migration, the migrant-receiving population can test many potential combinations between its existing lucky leaps and the newly arrived lucky leaps. Each of these potential combinations is useful with probability *P*_combUseful_, which we set equal to 1 in the following simulations to illustrate the potential scale of the effects of combining cultures. In reality, cultural trait combinations are not necessarily useful but are also not restricted to combinations of large-scale lucky leap innovations.

To consider separately the effects of migration and the effects of changes in population size, we assume that a migration event occurs according to a Moran model [47], with no change in size of either population: the migrant can be thought of as replacing a randomly chosen individual who died in the population it is joining, and its place in its original population is filled by a newborn individual. Lucky leap innovations that occur independently in different populations are assumed to be distinct, so two populations will initially have no tools in common. An individual from population 2 who joins population 1 brings population 2's lucky leap innovations and their associated toolkits. Once this occurs, a lucky leap that originated in population 2 can be combined with lucky leaps in population 1. For example, combining tool *A* from population 1 with tool *B* that originated in population 2 would lead to the combined tool *AB*. Also, all combinations of innovations *A* and *B* are identical to one another, even if the process that combined them occurred independently in different populations or occurred in a different order (*BA* = *AB*).

In human history, fortuitous innovations enabled increases in carrying capacity, resulting in population growth [30,31], which then likely facilitated larger cultural repertoires. We include this possibility in our model: with probability *P*_IncreaseCarryingCapacity_, each new combination increases the biological carrying capacity of the population. If this stochastic event occurs at time *t*, the population size (*N*) increases by a factor of *C*, sampled from a uniform distribution *U*(1.1,1.2): *N_t_*_+1_ = *N_t_* · *C*. As *P*_IncreaseCarryingCapacity_ acts on each new combination, carrying-capacity-altering traits are likely to arise when two cultures are connected by migration. We hypothesize that carrying-capacity-altering traits are resistant to cultural loss because the effects of losing the behaviour (for example, less available food) will quickly put pressure on the population to reintroduce it. Thus, carrying-capacity-altering combinations are placed in a distinct category of tools with their own loss probability, which we set to zero in the results presented below.

## 3. Results

### 3.1. The effect of population size on the cultural repertoire

As each individual may invent a novel tool with some probability, the rate of tool accumulation increases with population size. This accords with most models of cultural evolution, despite different approaches [12,37,39–41,46,48]. Loss of tools in our model is not implemented explicitly as a result of failed cultural transmission. To approximate tool loss, we implement directly the main qualitative finding of previous models with explicit transmission processes: a tool's probability of loss is inversely dependent on the population size, because additional tool users decrease the likelihood of failed transmission [28,36,38]. The combination of innovation and loss in our model leads to a nonlinear relationship between repertoire size and population size (equations (2.1)–(2.3)): repertoire size scales with *N*^2^ for lucky leap and toolkit innovations and with *N*^4^ for combination innovations. Slight variations of our model, such as different combination rules, would lead to somewhat different relationships between population size and repertoire size, but, qualitatively, the expected correlation is polynomial in *N* (see also [28]).

Because of this nonlinear dependence on population size, population subdivision has a dramatic effect on cultural repertoire size: a population of size 2*N* has a much higher cultural steady state in our model than the sum of two populations of size *N* (figure 1). As the relationship between *N* and repertoire size is highly sensitive to the details of the model, which is inevitably a gross simplification of reality, we do not attempt to fit our model's numerical results to empirical data. However, linking the qualitative trends produced by our model with empirical findings can be useful. Our model's prediction of a *polynomial* dependency of repertoire size on *N* implies that small differences in population size or connectivity can lead to previously underappreciated *disproportionate differences* in cultural complexity. This may help explain features of the transition from the Middle to the Upper Palaeolithic, as elaborated below.

### 3.2. The effect of rare migration on the cultural repertoire

In our model, combining existing tools can produce innovations. As a result, many new combinations are suddenly possible after an initial migration event, and testing these new combinations leads to a rapid burst of innovations. However, as we assume that the population size remains constant after migration [47], the cultural steady state is also constant. Thus, after the initial burst of innovation, the cultural repertoire gradually decays to approach the original steady state (figure 2).

### 3.3. The effect of frequent migration on the cultural repertoire

As the migration rate, *P*_migrate_, increases, the cultural repertoire of the receiving population does not have enough time between migration events to decay to the steady state (figure 3); thus, average cultural repertoire size increases. The effect of migration on the cultural steady state becomes more apparent as the migration rate increases: with more frequent migration, bursts of cultural accumulation no longer occur and a population has a relatively stable cultural repertoire that is substantially larger than the steady state predicted by its population size. This result accords with the findings of Powell *et al*. [12] regarding possible differences between world regions. Notably, in our model frequent migration between two populations of size *N* produces a total cultural repertoire that is smaller than that of an unstructured population of size 2*N* because the loss parameter, *P*_SpontLoss_, is still scaled by *N* and not 2*N*.

### 3.4. Migration and carrying capacity changes

In our model, following migration, tools that arose in separate populations can combine, and each may, with some probability, be a tool that increases carrying capacity. When this happens, the cultural steady state also increases, leading to more step-wise accumulation of culture (figure 4*a*) instead of a burst-and-decay pattern (figure 2). Carrying-capacity-altering innovations could also initiate a feedback loop: when the carrying capacity changes, the population grows and both the cultural repertoire and the effective migration rate increase, which further increases the likelihood that other carrying-capacity-altering innovations occur, ratcheting the cultural steady state upward (figure 4*b*).

Interestingly, if migration is intermediate in frequency, populations may evolve while remaining culturally distinct: core technologies are transmitted, but without the combination tools that are associated with each, and cultural losses are stochastic; thus, the combinations that arise in different populations only partially overlap. Major innovations, such as those that increase carrying capacity, are very likely to spread between the populations and remain shared, because of their adaptive value (our model implements this via the assumption that carrying-capacity-altering innovations are not stochastically lost; see also [5,12]). These shared carrying capacity increases lead to populations of similar size and thus similar cultural complexity (equations (2.1)–(2.3)). For a while, separate populations could co-develop: changes in population sizes and cultural complexity would occur separately in each population, but with higher correlation in timing than expected for independent populations (figure 5*a–c*). Populations would only remain separate temporarily: because population growth increases overall migration rate, eventually migration occurs frequently enough to prevent significant differentiation between the cultures (figure 5*d*). Note that *P*_migrate_, an individual's migration probability, changes in figure 5 at predetermined time steps, demonstrating the possible effect of sudden changes in migration rate.

## 4. Discussion

Human innovation is a multi-faceted process [42], but most models of cultural evolution primarily consider the transmission of existing cultural traits. To address this, we have proposed models that assess the role of interdependent innovation processes in causing cultural accumulation within a population [28,29]. However, recent experimental evidence underscores the importance of innovation via population interaction: groups with independently evolving cultural repertoires can produce useful new innovations by combining their existing innovations [15].

Here, we propose a fairly simple model that synthesizes these two research areas: multiple-independent populations undergo processes of innovation and cultural accumulation separately, and migration allows the populations’ cultural repertoires to be combined, producing additional innovations. Further, we consider that some of these novel cultural combinations might alter the biological carrying capacity of the population, causing population growth and a resulting increase in the cultural steady state, the population's expected number of cultural traits.

Our model produces five prominent patterns that appear to differ from those of most previous model-based studies. (i) We observe a polynomial relation between population size and cultural complexity, causing small changes in *N* to have disproportionate effects on repertoire size. (ii) We find that rare migration may lead to transient emergence of cultural complexity, which subsequently decays in small or relatively disconnected populations. (iii) Changes in migration rates may increase effective cultural population sizes with no change in local population sizes, potentially driving changes in cultural complexity. (iv) If culture affects carrying capacity or range expansion and if population size influences migration, a positive feedback loop may develop in which population growth, inter-population contact and cultural complexity interact. Such a feedback loop could have driven the demographic and cultural explosion that occurred in Eurasia shortly after the Middle to Upper Palaeolithic transition, as these three components are prominent characteristics of this transition [1,2,49–53]. Cultural innovations, such as inventions that change subsistence patterns or facilitate expansion to previously uninhabitable climates, could have driven population increases; support for both elements is found in the archaeological record [2,54–61]. (v) Complex cultural patterns may arise when multiple populations interact and exchange knowledge at intermediate frequencies, potentially driving one another towards related, but non-identical, trajectories of population growth and increased cultural complexity. These dynamics are transient if they subsequently increase migration, which eventually links the populations and homogenizes their cultures.

A single narrative is unlikely to accommodate the full range of archaeological observations regarding cultural evolution in the Palaeolithic. Instead, we propose that the patterns derived from our model may contribute to attempts to understand the archaeological record. By predicting a nonlinear relationship between population size and cultural repertoire, our model raises the possibility that undetectable increases in population size could drive disproportionately large changes in cultural complexity; alternatively, an increase in connectivity among populations, without population growth, could increase effective cultural population size and lead to cultural transition.

For example, although anatomically modern humans evolved in Africa approximately 160–200 ka [62–64], behavioural modernity occurred significantly later, with the full ‘package’ of cultural traits characteristic of the Upper Palaeolithic occurring only approximately 45 ka in the Levant, Europe, Western Asia and perhaps East/South Africa [2,65–69]. Estimates based on genetic and archaeological evidence indicate that both population sizes and densities increased in these regions near this time [49,70]. Our model predicts a nonlinear relationship between population size and cultural complexity, which suggests that cultural evolutionary dynamics could have driven the transition to behavioural modernity; thus, invoking biological change to explain this transition (as in [71,72]) is unnecessary. Qualitatively similar results and interpretations, relating cultural complexity to population size and migration in the transition to the Upper Palaeolithic, have been suggested by Powell *et al.* [12]. In addition, archaeological evidence does not unequivocally support significant population growth in Africa 50–45 ka [73,74], which has generated criticism towards attributing behavioural modernity's emergence to population size [11,75,76]. Our model's prediction that small changes in population sizes or migration patterns could drive large cultural change may contribute to this discussion. Further, a major characteristic of the Upper Palaeolithic revolution is the dramatic increase in the distance of material and artefact transportation [1,2,77,78]. As an increase in contact can effectively connect populations, thus forming a single meta-population with a larger cultural repertoire, our results suggest that connectivity could have been a major *driver* of this cultural revolution and not just one of its *outcomes* (see also [43], which analyses the combined effect of connectedness with demographic fluctuation and local extinction).

A nonlinear relationship between population size and cultural complexity also provides a possible explanation for the occurrence of full behavioural modernity only among modern humans: estimates from genetic diversity suggest that Neanderthals had a threefold smaller effective population size than modern humans [79–81]. Neanderthals and modern humans may have had similar cognitive and physical capacity for behavioural modernity [82–85], yet behavioural modernity only occurs in humans following the Neanderthal replacement [52,86].

In our model, when an individual migrates to a new population, the receiving population experiences a cultural burst because many novel combinations of innovations are suddenly possible. However, when migration is very rare, the population size, and thus the cultural steady state, remains constant, and the receiving population experiences a gradual decay to its original steady-state repertoire size (figure 2). This decay in the cultural repertoire after the initial acquisition of imported knowledge has precedents in the anthropological literature: even beneficial cultural traits from one population do not necessarily spread in another [87,88], potentially because of conflicting cultural norms [88] or language barriers [89]. Our model demonstrates that these complex cultural dynamics might occur, without making assumptions about social networks or transmission rules.

A population's migration rate may depend on numerous factors, including geographical boundaries, subsistence strategies and cultural practices, which may help explain the patchy appearance and disappearance of stone tool techno-complexes and other cultural practices during the Lower and Middle Palaeolithic [2,18,90]. More frequent contact between two populations would effectively increase the tool repertoire at steady state, as migrants may reintroduce cultural traits before the receiving population's repertoire can fully decay to its steady state (figure 3). Migration can thus foster an elevated level of culture, either because migration occurs regularly or because the cultural steady state increases by some other mechanism.

We explore one such mechanism by considering cultural traits that alter the biological carrying capacity. Throughout history and prehistory, cultural innovations have enabled human populations to extract more resources from their habitat, probably leading to population growth and subsequent increase of the cultural repertoire. In our model, when independent cultures come into contact, we assume that with small probability an innovative combination of their traits will increase the biological carrying capacity. The increased carrying capacity allows population growth, in turn elevating the cultural steady state. Thus, after a carrying-capacity-altering innovation occurs, the contact-induced burst of innovative combinations persists instead of decaying (figure 4).

The results in figure 5 provide a possible explanation for one of the hotly debated observations in the transition between the Middle and Upper Palaeolithic in Europe: the transient prosperity of many cultures within a relatively short time span near the Middle–Upper Palaeolithic transition, such as the Uluzzian, Bachokirian, Châtelperronian, Bohunician and Proto-Aurignacian [91–95], which were distinct yet shared a number of characteristics that set them all apart from Middle Palaeolithic cultures [51,86,94,96–102]. This relatively sudden appearance of multiple distinct complex cultures with shared features is unlikely to be a coincidence. It could have been brought about by gradual diffusion of core technologies via rare migration, creating an increase in cultural complexity, which was coordinated among multiple localities yet rare enough to maintain differences between them, as seen in figure 5 (*t* < 500). Our model proposes that, with time, cultural evolution could have affected population sizes and, as a result, the migration rates between them, leading to decreasing cultural differentiation between local populations (figure 5, *t* > 500). The prehistoric record in Eurasia is characterized by a similar pattern: an increase in rates of population interaction in the Upper Palaeolithic [1,2,77,78], driven by population growth or by behavioural change, and replacement of the multitude of techno-complexes near the Middle–Upper Palaeolithic transition by the Aurignacian [86,96,97,99].

Another conspicuous archaeological pattern is the sporadic transient appearance of ‘advanced’ behaviours, characteristic of the Upper Palaeolithic (Late Stone Age in Africa), well within the Middle Stone Age: these include evidence of abstract art such as engraved ochre pieces and incised ostrich eggs, personal ornamentation such as shell beads, and advanced bone and stone tools [2,62,103–111]. A possible explanation of the transient nature of these phenomena is that, as in our simulations, the populations in which they occurred were too small and disconnected from one another to stably maintain complex culture (see also [18]).

Many large-scale cultural shifts have been attributed to external factors, such as environmental change and resource availability [23,112,113], or cognitive and genetic changes [21,25–27]; in these examples, non-cultural changes facilitate a cultural response, resulting in increased cultural accumulation. Here, we have explored two *cultural* factors that can provoke bursts of innovation: population contact via migration, and modification of the biological carrying capacity. A recent archaeological study [114] suggested that large cultural changes facilitate human expansion to new areas. Building on this idea, migration could introduce new information to a population, leading to range expansion, which could be another sense in which cultural changes could generate population growth. This raises a direction of causality question in interpreting the Palaeolithic revolution: did increased migration bring about cultural bursts, leading to increased carrying capacities and resulting growth across populations? Or did a carrying-capacity-modifying innovation occur in one population, which in turn brought about cultural changes that subsequently facilitated migration, expansion and population growth?

## Supplementary Material

Model description and analytical predictions

## Data accessibility

The simulation code is available at https://github.com/CreanzaLab/CulturalMigrationAndConnectivity.
