# Supplementary material for: Greater than the sum of its parts? Modelling population contact and interaction of cultural repertoires
Source: J R Soc Interface. 2017 May 3;14(130):20170171. doi: 10.1098/rsif.2017.0171 (PMC5454306; doi:10.1098/rsif.2017.0171)
Supplement: Model description and analytical predictions [file rsif20170171supp1.pdf]

Electronic supplementary material: Model description and analytical predictions

Greater than the sum of its parts? Modeling population contact and interaction of cultural repertoires

Nicole Creanza, Oren Kolodny, & Marc Feldman

This paper extends the model of Kolodny *et al*. [1] to include multiple populations that can independently undergo processes of innovation and cultural evolution, and to explore the effects of migration between these populations, simulating the outcomes of population contact and cultural interactions. As in [1], three interacting processes contribute to human innovation of tools; each of these occurs with a different probability in an agent-based stochastic simulation. The first process produces groundbreaking large-scale innovations, or *lucky leaps*. Once such an innovation exists in a population, it can facilitate the other two tool innovation processes. First, a number of tools are made useful by each lucky leap; these are termed *toolkit innovations*. Second, lucky leap innovations can also combine with other lucky leap innovations to produce *innovative combinations*. Finally, a cultural trait can be stochastically lost at a rate that is inversely proportional to the number of individuals in a population who have the trait.

Here, we describe the processes involved in the model in detail, and we give equations for the *expected* effect of each process. The equations presented here are approximations that provide intuition for each process, but each run of the stochastic simulation is unique. Agent-based simulations with stochastic processes, such as this one, often do not allow simple analytical descriptions of their dynamics. Here, we give

equations for the expected effect of each of the innovation processes under some simplifying assumptions, and we provide an analytical explanation for equations (1)–(3) in the main text, based on the relevant sections of the Electronic Supplemental Materials of Kolodny et al. 2015 [1].

With probability P_{lucky} per individual, lucky leaps are added at each time step to the cultural repertoire of a population of size N, leading to an expected rate of change in the number of lucky leaps

$$\frac{\Delta n_{lucky}}{\Delta t} = P_{lucky} \cdot N \,. \tag{1a}$$

In a population of N individuals that at time t=0 has a cultural repertoire of size zero, the expected number of lucky leap innovations at time t that have been produced in this process, n_{lucky}, is thus:

$$n_{lucky} = P_{lucky} \cdot N \cdot t \tag{1b}$$

In our simulations, tools can be lost as a result of stochastic drift. This stochastic loss occurs at a rate that is dependent on the number of existing tools and inversely proportional to the population size. If we consider only lucky leaps, the rate of spontaneous loss from the tool repertoire is:

$$\frac{\Delta n_{lucky}}{\Delta t} = \frac{\mathrm{P}_{\mathrm{SpontLoss}}}{N} \cdot n_{lucky} \,. \tag{2}$$

Subtracting this loss term from the rate of accumulation of lucky leaps given in equation 1a, we get:

$$\frac{\Delta n_{lucky}}{\Delta t} = P_{lucky} \cdot N - \frac{\mathrm{P}_{\mathrm{SpontLoss}}}{N} \cdot n_{lucky} \,. \tag{3}$$

We assume that the population has no tools at time t=0 (n_0=0), and we treat this difference equation as a continuous time differential equation with solution at time t:

$$n_{lucky} = \frac{N^2 \cdot P_{lucky}}{P_{SpontLoss}} - \frac{N^2 \cdot P_{lucky}}{P_{SpontLoss}} \cdot e^{\frac{-P_{SpontLoss}}{N} \cdot t} \quad (4)$$

At large t, the curve described by this function approaches an asymptote:

$$n^*_{lucky} = \frac{N^2 \cdot P_{lucky}}{P_{SpontLoss}}. \quad (5)$$

This is Equation 1 in the main text. Here we have made the simplifying assumption that loss of a tool is final, i.e. that reinvention of the same tool does not occur. In the stochastic simulations, however, this applies to lucky leaps but not to toolkit tools or combination innovations. If a toolkit or combination tool is lost but the lucky leap associated with it remains in the cultural repertoire, the population is capable of reinventing the lost toolkit or combination tool.

We assume that there are L toolkit innovations associated with each lucky leap, where L is sampled from a uniform distribution U(1,11). Then, if $\langle L \rangle$ is the expected value of L (namely 5.5), the expected rate of change of toolkit innovations is $\frac{\Delta n_{toolkit}}{\Delta t} = P_{lucky} \cdot N \cdot \langle L \rangle$, provided the L toolkit innovations associated with a lucky leap tool are invented immediately following the lucky leap innovation. This assumption is a reasonable approximation of the stochastic dynamics when the rate of toolkit innovation is orders of magnitude higher than the rate of lucky leap innovations. Combining the expected rates of change of lucky leaps and toolkit innovations, a population of N

individuals thus has an expected number of lucky leaps (n_{lucky}) and toolkit innovations ($n_{toolkit}$) at time t given by:

$$n_{lucky} + n_{toolkit} = P_{lucky} \cdot N \cdot t + P_{lucky} \cdot \langle L \rangle \cdot N \cdot t. \tag{6}$$

When lucky leap innovations are combined to produce *innovative combinations*, these combinations are useful to the population with probability $P_{CombUseful}$, leading to an expected rate of change of $\frac{\Delta n_{comb}}{\Delta t} = P_{lucky} \cdot N \cdot n_{lucky} \cdot P_{CombUseful}$, where n_{comb} is the expected number of innovative combinations and n_{lucky} is defined in equation (1). As with toolkit innovations, we make the simplifying assumption that all combinations with a given lucky leap are tested immediately following that lucky leap's innovation. When innovations can combine in this way to form new tools, the expected size of the tool repertoire of a population of size N at time t is $n_{total} = n_{lucky} + n_{toolkit} + n_{comb}$:

$$n_{total} = P_{lucky} \cdot N \cdot t + P_{lucky} \cdot \langle L \rangle \cdot N \cdot t + \frac{\left(P_{lucky} \cdot N\right)^2}{2} \cdot P_{CombUseful} \cdot t^2 \ . \tag{7}$$

This combination scheme represents the notion that groundbreaking ideas are often widely applicable to other existing technologies, and it is relatively conservative since only lucky leaps can be combined to produce new innovations. For simplicity we assume that all potentially useful combinations and toolkit tools are innovated immediately upon the lucky leap's invention, that is, the population can produce all useful combinations of tools whenever a new tool becomes available, so that an individual may test more than one combination per time step.

Because the rate of cultural loss of tools is likely to decrease as population size increases (e.g. [2]), we assume that tools can be lost at each time step with probability

$P_{SpontLoss}/N$. Toolkit innovations and combination tools can be individually lost, but if a lucky leap tool is lost, the toolkit and combination tools associated with it are also lost. With probability $\frac{n_{lucky}}{n_{total}}$, the tool that is lost is a lucky leap, and thus its associated toolkit and combinations are lost with it, so when a lucky leap is lost, the total number of tools lost is $L_t + C_t + 1$, where L_t and C_t are, respectively, the mean number of toolkit innovations and combination innovations associated with a lucky leap innovation at the time of its loss, t, and the 1 accounts for the lucky leap itself. In our model, each combination tool is formally associated with only one of the lucky leaps from which it is composed, and thus the loss of that lucky leap leads to the loss of the combination. The Electronic Supplementary Material from [1] also examines the alternate assumption in which a combination tool is lost following the loss of either of its component lucky leaps, which does not qualitatively alter the results. With probability $1-\frac{n_{lucky}}{n_{total}}$, the tool lost is a toolkit or combination innovation, and the number of tools lost is 1. This leads to the difference equation:

$$\frac{\Delta n_{total}}{\Delta t} = P_{lucky}\cdot N + P_{lucky}\cdot N\cdot\langle L\rangle + P_{lucky}\cdot N\cdot\langle C_{inv}\rangle\cdot f - \frac{P_{SpontLoss}}{N}\cdot n_{total}\cdot\left(\frac{n_{lucky}}{n_{total}}\cdot\left(\langle L_t\rangle+\langle C_t\rangle+1\right)+\left(1-\frac{n_{lucky}}{n_{total}}\right)\right), \quad (8)$$

where C_{inv} is the average number of new useful combination tools that can be made possible by a new lucky leap upon its invention (which is dependent on the existing number of lucky leaps, n_{lucky}), and where $\langle C_{inv}\rangle$ is the expected value of C_{inv}.

The last term in (8) is the expected loss term for a population of size N to be subtracted from the expected number of innovations generated at time t. This loss term can be simplified, and replacing n_{total} by $n_{lucky} + n_{toolkit} + n_{comb}$, equals to:

$$-\frac{P_{SpontLoss}}{N}\cdot\left(n_{lucky}+n_{toolkit}+n_{comb}\right)-\frac{P_{SpontLoss}}{N}\cdot n_{lucky}\cdot\left\langle L_t\right\rangle-\frac{P_{SpontLoss}}{N}\cdot n_{lucky}\cdot\left\langle C_t\right\rangle \quad (8a)$$

Since $\left\langle C_{inv}\right\rangle$ and $\left\langle C_t\right\rangle$ change with time because of their dependence on (the time-dependent) n_{lucky}, the solution of equation (8) is complicated. Here, we calculate the steady-state value of the cultural repertoire for each type of tool separately.

To find the asymptote approached for each type of tool, we separate equation (8) into three equations representing the rate of change of each distinct type of tool. When the cultural repertoire is at equilibrium, the number of lucky leaps, toolkit tools, and combination innovations do not change. Thus, we can set the rates of change $\frac{\Delta n_{lucky}}{\Delta t}$, $\frac{\Delta n_{toolkit}}{\Delta t}$, and $\frac{\Delta n_{comb}}{\Delta t}$ equal to zero. Separating equation 8 into three equations by type of tool is most intuitive using the loss term as written in expression 8a. As noted earlier, the first of the three equations is, from equation 3, $\frac{\Delta n_{lucky}}{\Delta t}=P_{lucky}\cdot N-\frac{\mathrm{P}_{\mathrm{SpontLoss}}}{N}\cdot n_{lucky}$, and leads to a steady-state value of $n^{*}_{lucky}=\frac{N^2\cdot P'_{lucky}}{P_{SpontLoss}}$ (5, Equation 1 in the main text).

The second is:

$$\frac{\Delta n_{toolkit}}{\Delta t}=P_{lucky}\cdot N\cdot\left\langle L\right\rangle-\frac{\mathrm{P}_{\mathrm{SpontLoss}}}{N}\cdot n_{toolkit}-\frac{P_{SpontLoss}}{N}\cdot n_{lucky}\cdot\left\langle L_t\right\rangle \quad (9)$$

where $-\frac{P_{SpontLoss}}{N} \cdot n_{toolkit}$ accounts for direct loss of toolkit innovations and $-\frac{P_{SpontLoss}}{N} \cdot n_{lucky} \cdot \langle L_t \rangle$ accounts for the loss of toolkit tools that were associated with a lucky leap that was itself lost. The mean size of a lucky leap's toolkit at the time of its loss is $\frac{\langle L \rangle}{2}$, because individual toolkit tools and lucky leaps are lost with equal probability, so, on average, half of the toolkit innovations will be lost by the time the lucky leap associated with them is lost. Using this and the steady state for lucky leaps in equation 5, we find

$$n^*_{toolkit} = \frac{N^2 \cdot P_{lucky} \cdot \langle L \rangle}{2 \cdot P_{SpontLoss}} . \qquad \text{(10, Equation 2 in the main text)}$$

Similarly, the third equation is

$$\frac{\Delta n_{comb}}{\Delta t} = P_{lucky} \cdot N \cdot \langle C_{inv} \rangle \cdot P_{CombUseful} - \frac{P_{SpontLoss}}{N} \cdot n_{comb} - \frac{P_{SpontLoss}}{N} \cdot n_{lucky} \cdot \langle C_t \rangle , \qquad (11)$$

with $\langle C_t \rangle = \frac{\langle C_{inv} \rangle}{2}$.

Since combination tools are made up of a new lucky leap and the lucky leaps already in the cultural repertoire, when a lucky leap occurs in a population at steady state, the average number of combination tools that turn out to be useful is $\langle C_{inv} \rangle = P_{CombUseful} \cdot n^*_{lucky}$. Using the expression for this term, we find

$$n^*_{comb} = \frac{N^4 \cdot \left(P_{lucky}\right)^2 \cdot P_{CombUseful}}{2 \cdot \left(P_{SpontLoss}\right)^2} . \qquad \text{(12, Equation 3 in the main text)}$$

References

1. Kolodny, O., Creanza, N. & Feldman, M. W. 2015 Evolution in leaps: The punctuated accumulation and loss of cultural innovations. *Proc. Natl. Acad. Sci.* **112**, E6762–E6769. (doi:10.1073/pnas.1520492112)

2. Henrich, J. 2004 Demography and Cultural Evolution: How Adaptive Cultural Processes can Produce Maladaptive Losses: The Tasmanian Case. *Am. Antiq.* **69**, 197–214.
